# Deformability-Based Isolation of Circulating Tumor Cells in Spiral Microchannels

**DOI:** 10.3390/mi14112111

**Published:** 2023-11-17

**Authors:** Roya Mohammadali, Morteza Bayareh

**Affiliations:** Department of Mechanical Engineering, Shahrekord University, Shahrekord 88186-34141, Iran; mohamadali.r28@gmail.com

**Keywords:** microfluidics, cell separation, CTC, deformability

## Abstract

The isolation of circulating tumor cells (CTCs) and their analysis are crucial for the preliminary identification of invasive cancer. One of the effective properties that can be utilized to isolate CTCs is their deformability. In this paper, inertial-based spiral microchannels with various numbers of loops are employed to sort deformable CTCs using the finite element method (FEM) and an arbitrary Lagrangian–Eulerian (ALE) approach. The influences of cell deformability, cell size, number of loops, and channel depth on the hydrodynamic behavior of CTCs are discussed. The results demonstrate that the trajectory of cells is affected by the above factors when passing through the spiral channel. This approach can be utilized for sorting and isolating label-free deformable biological cells at large scales in clinical systems.

## 1. Introduction

The isolation of rare cells, including CTCs, is a complicated process because they are notably unprotected from unintended losses [1]. The isolation of single cells from heterogeneous samples is pivotal in medical and biological applications [2,3]. On the other hand, the deformability or mechanical stiffness of individual cells affects the cellular phenotype in biological assessments [4]. It has been demonstrated that the deformability of CTCs is excessively diminished in comparison with normal tissues and correlated with metastatic potential [5].

Two major criteria for isolating CTCs from blood cells are their size and deformability. The diameter of epithelial CTCs varies from 14 μm to 26 μm and the diameter of white blood cells (WBCs) changes from 8 μm to 20 μm. Various passive and active label-free techniques have been utilized to separate cells, such as hydrodynamic methods that isolate cells based on their size, filtration methods that separate cells due to their size and deformability, and inertial focusing by employing cell intrinsic characteristics, i.e., size and stiffness [6,7,8].

The influence of the inertial lift force, $F_{L}$, was first considered by Di Carlo et al. [9]. They assessed the trajectory of particles in straight and curved microchannels. It has been demonstrated that the channel Reynolds number, ${Re}_{ch}=U_{max}D_{h}/\nu$, and the particle Reynolds number, ${Re}_{p}={Re}_{ch}D^{2}/D_{h}^{2}$, where $U_{max}=1.5U_{avg}$, $D_{h}$, ν, and D are the maximum fluid velocity, hydraulic diameter, kinematic viscosity, and particle diameter, respectively, describe the impact of the inertial force on the motion of microparticles [10]. Di Carlo et al. [9] examined the influence of ${Re}_{p}$, $D/D_{h}$, the density of microparticles, etc., and suggested the optimal ranges of these factors to augment the impact of $F_{L}$ on particle focusing. Di Carlo et al. [11] also revealed that the optimal amount of flow rate for their proposed microfluidic device is 0.9 μL/min to isolate platelets from whole blood. Ozkumur et al. [12] carried out experimental examinations to sort various kinds of CTCs, including prostate, breast, pancreas, melanoma, and lung, utilizing inertial force. Their microfluidics device involved three outlets. Red blood cells (RBCs), platelets, and other blood components exited from the first outlet. Then, CTCs labeled with magnetic beads and WBCs were sorted based on their size and deformability in a curved channel. They were then separated by applying a magnetic field and exited from the second and third outlets. Oakey et al. [13] introduced a staged microchannel consisting of straight and curved sections for ${0.2\leq Re}_{p}\leq 5.9$. The curved section was the same as the one utilized by Ozkumur et al. [12]. The effectiveness of their proposed device was augmented by enhancing the fluid throughput and particle concentration. Kim et al. [14] carried out a parametric study to evaluate the impact of inertial focusing in a curved microchannel on the separation of CTCs. The effective parameters included the height and width of the microchannel as well as the curvature radius. Using sheath-less spiral microchannels, they isolated human breast cancer cells from WBCs with a purity of 86.76%. Abdulla et al. [15] employed a cascade inertial focusing device consisting of two spiral microchannels and a zigzag one to separate lung and breast cancer cells from WBCs and reached cell viability > 95%.

Almost all numerical simulations have not examined the deformability of CTCs during their continuous separation because the stiffness can be considered by utilizing specific numerical techniques, such as fluid-solid interaction (FSI), for individual cells. Hence, numerical works have been limited to size-based continuous separation of CTCs. For example, Ozbey et al. [16] employed COMSOL multiphysics software 6.1 to assess the focusing treatment of four types of CTCs utilizing a curvilinear geometry. It was found that 11–22 μm CTCs can be isolated from 8–17 μm CTCs when the curvature angle is 280° and ${Re}_{ch}$ = 121. Shiriny and Bayareh [17] utilized a single-loop spiral microfluidic device to isolate breast and epithelial cervical cancer cells and reported that the separation efficiency may reach 100% for ${90\leq Re}_{ch}\leq 110$. Recently, Pakhira et al. [18] carried out three-dimensional simulations to isolate various kinds of CTCs, such as breast, prostate, and lung cells, using inertial-based single- and double-loop spiral microchannels for ${55.9\leq Re}_{ch}\leq 65$. It was revealed that the single-loop device is more efficient than the double-loop one.

Even though several experimental studies have proposed deformability-based inertial devices [19,20], a few numerical works have assessed deformability influences on the focusing behavior of CTCs in different microfluidic geometries. For instance, Quek et al. [21] utilized the immersed boundary scheme to separate deformable cells in deterministic lateral displacement (DLD) devices consisting of regular arrays of microposts. They reported three types of cell trajectories, including zigzag, laterally displaced, and dispersive. The third type was observed for large and rigid particles when their stiffness was > 500 MPa. Additionally, Mohammadali et al. [22] employed the FSI approach to predict the impact of deformability on the trajectory of WBCs and two types of CTCs in a DLD device. They demonstrated that deformability influence on the trajectory of soft cells is higher than stiff ones, especially at low amounts of ${Re}_{ch}$. It was also revealed that I-shaped micropillars lead to larger lateral displacement of CTCs. Guzniczak et al. [23] assessed the hydrodynamic behavior of cells in a spiral channel and demonstrated that equal-sized cells have different equilibrium positions according to their deformability. It was reported that the final position of soft and stiff cells is in the vicinity of the outer and inner walls, respectively.

**Figure 1:**
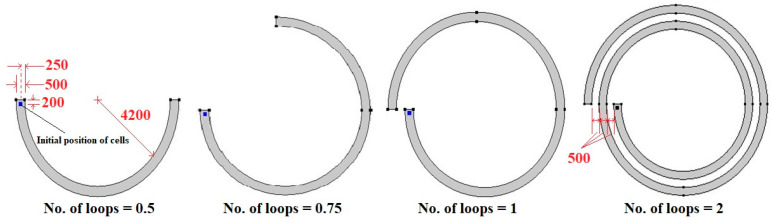
Schematic of spiral microchannels with various numbers of loops (all the dimensions are in micrometers).

In the present paper, the FSI approach is employed for the first time to evaluate the impact of deformability on the trajectory of WBCs and two kinds of CTCs in spiral microchannels. The hydrodynamic behavior of soft and stiff cells of various sizes is studied when the number of loops is 0.5, 0.75, 1, and 2 (Figure 1). This approach can be utilized for sorting and isolating label-free deformable biological cells at large scales in clinical systems. The rest of the paper is organized as follows. The deformable particle theory and mathematical model are presented in Section 2. Section 3 provides boundary and initial conditions. Grid study and validation are provided in Section 4. Section 5 discusses the numerical results, and concluding remarks are presented in Section 6.

## 2. Theoretical Background

### 2.1. Fluid Flow

As Figure 1 demonstrates, spiral microfluidic devices with various numbers of loops are considered. A spherical WBC or CTC with different radiuses is located at a distance of 200 μm from the channel inlet and a distance of 250 μm from the channel walls. Since the blood is diluted for real applications, the number of RBCs per volume is reduced considerably; hence, it can be assumed to be a Newtonian fluid. The governing equations, i.e., continuity and Navier–Stokes equations, for Newtonian incompressible fluid are expressed as follows [17]:

(1) $$\nabla \cdot u_{f}=0$$

(2) $$\rho_{f}\frac{\partial u_{f}}{\partial t}+\rho_{f}\left(u_{f}\cdot \nabla \right)u_{f}=\nabla \cdot \left[-p_{f}I+\mu_{f}\left(\nabla u_{f}+{\left(\nabla u_{f}\right)}^{T}\right)\right]+F_{f}$$

where $u_{f}$ and $p_{f}$ represent fluid velocity vector and pressure, respectively. The density and dynamic viscosity of fluid are $\rho_{f}$ = 1000 $kg/m^{3}$ and $\mu_{f}$ = 0.001 Pa.s, respectively. Since volume forces, such as gravitational force, are not applied in the present simulation, $F_{f}=0$.

For rigid particles, spiral microchannels work based on the balance between the inertial lift force ($F_{L}$) and Dean drag force ($F_{D}$) [24,25]:

(3) $$F_{L}=\frac{\rho U_{max}^{2}r_{P}^{4}}{D_{h}^{2}}C_{L}$$

(4) $$F_{D}\approx 5.4\times 10^{-4}\pi \mu {De}^{1.63}r_{P}$$

where $r_{P}$ and $C_{L}$ are the particle radius and lift coefficient, respectively. $De={Re}_{ch}\sqrt{D_{h}/2R}$ represents Dean number, where R is the radius of the channel curvature. Thus, $F_{s}=F_{L}+F_{D}$ for rigid cells.

For deformable particles moving in straight [8] and curved [23] microchannels, an additional lift force, named deformability-induced lift force $F_{D-L}$, affects their migration. Guzniczak et al. [23] examined the contribution of $F_{D-L}$ to the migration mechanism of soft particles in a microchannel with a cross-section of 360 × 60 μm^2^. They concluded that the impact of $F_{D-L}$ can be neglected when ${Re}_{ch}$ is low. It was revealed that the lateral equilibrium position of deformable cells becomes closer to the outer wall of a curved microchannel due to the influence of $F_{D-L}$.

In the present work, $D_{h}=2WH/\left(W+H\right)$, where W and H denote the microchannel width and depth, respectively. The ratio of H/W determines the number of lateral focusing positions [8]. For continuous separation of particles, when H/W>2, the particle overlap possibility is diminished.

### 2.2. Solid Mechanics

Many microfluidic devices employ inertial forces to focus or sort cells using passive methods. The trajectory of deformable cells can be described as follows utilizing the governing equations of linear elastodynamics [24]:

(5) $$\rho_{s}\frac{\partial^{2}u_{s}}{\partial^{2}t}=F_{s}+\nabla \cdot \sigma_{s}$$

(6) $$\sigma_{s}=C\varepsilon_{s}$$

(7) $$\varepsilon_{s}=\frac{1}{2}[{\left(\nabla u_{s}\right)}^{T}+\nabla u_{s}+{\left(\nabla u_{s}\right)}^{T}(\nabla u_{s})]$$

Here, $u_{s}$, $\sigma_{s}$, $\varepsilon_{s}$, $\rho_{s}$, $F_{s}$, and C are the solid displacement field, Cauchy stress tensor, strain tensor, solid density, volumetric force per unit volume, and stiffness matrix, respectively. The stiffness matrix is defined as follows:

(8) C=Es1+Rs1−2Rs1−RsRsRs000Rs1−RsRs000RsRs1−Rs0000001−2Rs0000001−2Rs0000001−2Rs

Here, $E_{s}$ and $R_{s}$ represent Young’s modulus and the Poisson ratio of the solid, respectively. Equations (3)–(6) can be solved when the amounts of $\rho_{s}$, $F_{s}$, $E_{s}$, and $R_{s}$ are known. The density of CTCs and WBCs is assumed to be 1050 $kg/m^{3}$. The properties of cells are presented in Table 1.

### 2.3. FSI

Fluid flow and solid mechanics are coupled by the FSI to capture the interface between two phases. This approach describes the impact of fluid flow on the solid boundary and the influence of solid displacement on fluid velocity:

(9) $$f_{s}=-n\cdot \left[-p_{f}I+\mu_{f}\left(\nabla u_{f}+{\left(\nabla u_{f}\right)}^{T}\right)\right]$$

(10) $$u_{w}=\frac{\partial u_{s}}{\partial t},\;u_{w}=u_{f}$$

where $f_{s}$, n, and $u_{w}$ are the total force exerted on the solid boundary, the normal vector, and the change rate of $u_{s}$.

The present system is solved using the FEM and ALE method by employing COMSOL Multiphysics software. The ALE approach has been verified for deformable particles [27]. This technique combines the interface between the fluid and the solid.

The governing equations are discretized by the FEM using simple functions. A Lagrangian approach and a material frame are utilized to formulate the solid mechanics. Since the solid domain has a constant number of nodes, the following relation can be expressed between $f_{s}$ and $F_{s}$ [28]:

(11) $$F_{s}=f_{s}\frac{dv}{dV}$$

Here, dv and dV denote the scale factors for the grid elements of the spatial and material frames, respectively.

## 3. Boundary and Initial Conditions

The fluid flows in the spiral microchannel due to the pressure gradient between its inlet and outlet. The fluid flow is assumed to be fully developed and laminar and its parabolic velocity distribution is described as follows, where Y is the material frame coordinate along the microchannel inlet [28].

(12) $$U_{in}=U_{avg}\frac{6\left(W-Y\right)Y}{W^{2}}$$

The boundary condition at the outlet is the Dirichlet condition:

(13) $$p_{f}=0,\;\mu_{f}\left(\nabla u_{f}+{\left(\nabla u_{f}\right)}^{T}\right)n=0$$

Additionally, the no-slip boundary condition is also used for the microchannel walls:

(14) $$u_{f}=0$$

Also, the initial amounts of $u_{f}$, $p_{f}$, and $u_{s}$ are assumed to be zero for simplicity.

## 4. Grid Study and Validation

**Figure 2:**
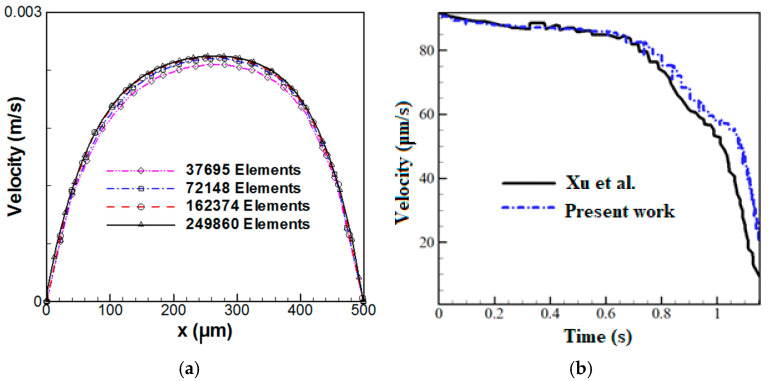
(**a**) Velocity distribution in a cross-section located at a 500 μm distance from the inlet for various grid resolutions and (**b**) microparticle velocity versus time: a comparison between the present numerical results and the experimental data of Xu et al. [28].

Due to the motion of cells, the grid is deformed during the simulations, resulting in a reduction in the grid quality when the cell moves through the microchannel. Thus, the computational domain is re-meshed each time to provide a grid quality below 0.7. Four grid resolutions are utilized to perform the grid independence test. Figure 2a illustrates the velocity distribution for a cross-sectional area located at a 500 μm distance from the inlet. Four grids with 37,695, 72,148, 162,374, and 249,860 elements are considered in this figure, indicating that the grids with 162,374 and 249,860 elements result in the same velocity profile. Thus, the grid with 162,374 elements can be selected for further simulations.

The present numerical results are compared and verified using the experimental data and numerical results of Xu et al. [28]. For numerical simulations, they utilized the FEM to evaluate the hydrodynamic trapping of deformable particles in a microfluidic device consisting of microposts. Figure 2b illustrates the variations of microparticle velocity as a function of time, indicating a good agreement between the present results and experimental data.

## 5. Results and Discussion

In this section, the isolating process of single cells is examined based on their size and deformability. The influences of cell stiffness, cell size, the number of loops, and microchannel depth on cell trajectory are examined.

### 5.1. Effect of Cell Deformability

**Figure 3:**
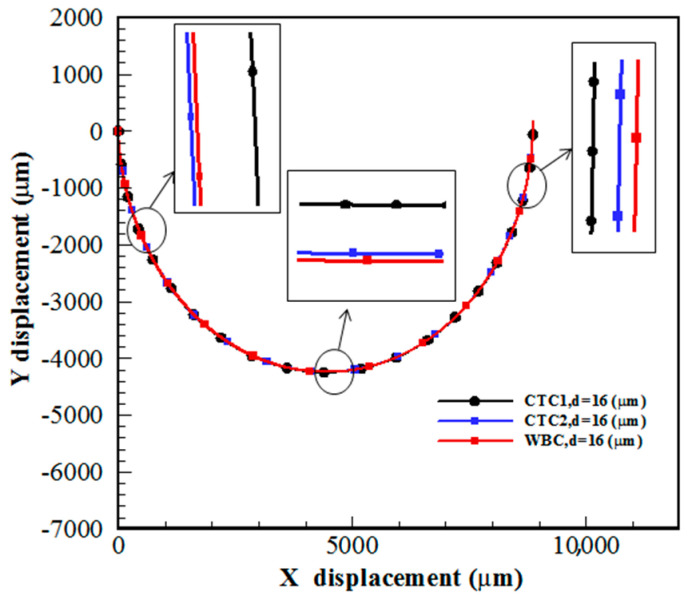
Trajectory of CTC1, CTC2, and WBCs in a 0.5-loop spiral microchannel when ${Re}_{ch}=1$.

**Figure 4:**
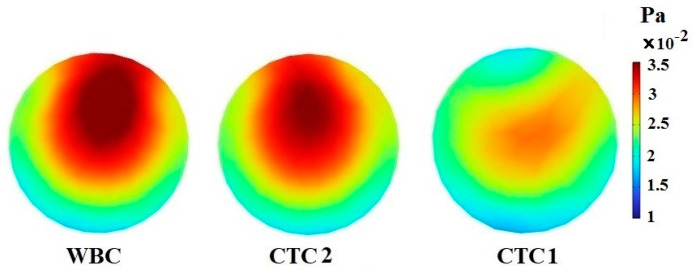
The stress contour inside CTC1, CTC2, and WBCs when they reach the end of the 0.5-loop spiral microchannel.

The most important reason for the separation of CTCs is the formation of Dean flow in spiral channels, leading to the creation of a secondary flow in the channel cross-section. In this section, CTC1, CTC2, and WBCs with the same diameter of 16 μm enter the spiral microchannel along with the diluted blood flow when ${Re}_{ch}=1$. Cells are subjected to lift force in the direction perpendicular to the flow and drag force in the flow direction. Due to the small size of the cells, the gravity force is neglected. As mentioned previously, deformable cells also experience an additional lift force $F_{D-L}$, which pushes them to an equilibrium position closer to the outer wall of the channel, while rigid particles are concentrated closer to the inner wall. For a constant cell size, deformability has a great effect on the trajectory of CTCs. Figure 3 demonstrates that the cells tend to move toward the outer wall as deformability is augmented. Softer cells are more deformed due to pressure and shear stress (Figure 4). In other words, CTCs show higher stress than WBCs due to their larger size and various mechanical characteristics. Additionally, the stress distribution indicates a core-like pattern in CTCs and WBCs that can be utilized to design inertial-based microfluidic separation devices.

### 5.2. Effect of Cell Size

**Figure 5:**
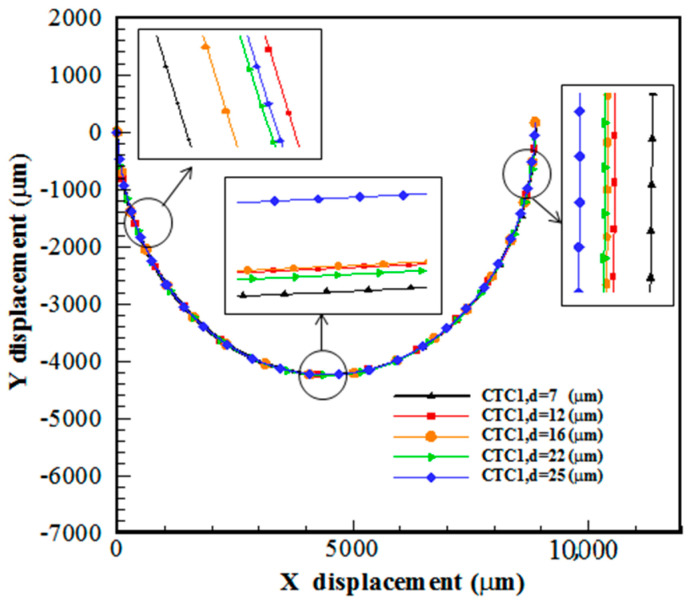
Trajectory of CTC1 of various sizes in a 0.5-loop spiral microchannel when ${Re}_{ch}=1$.

In a spiral microchannel, the deformable cell equilibrium position is determined due to the balance between $F_{L}$, $F_{D}$, and $F_{D-L}$. The cells with different sizes experience various hydrodynamic behaviors due to the formation of Dean flow. As shown in Figure 5, larger cells are directed toward the inner channel wall. Smaller cells follow the direction of $F_{D}$, i.e., the secondary flow, and move toward the outer channel wall, which is consistent with the experimental results of Guan et al. [29]. The Dean vortices in the vicinity of the walls are weak and cannot re-entrain smaller cells. This difference in the focusing behavior of large and small cells provides an opportunity for their separation. In other words, larger cells enter the Dean flow due to the influence of lift and drag forces; however, smaller ones do not remain in Dean vortices due to not overcoming the centrifugal force and move in the outer part of the microchannel.

**Figure 6:**
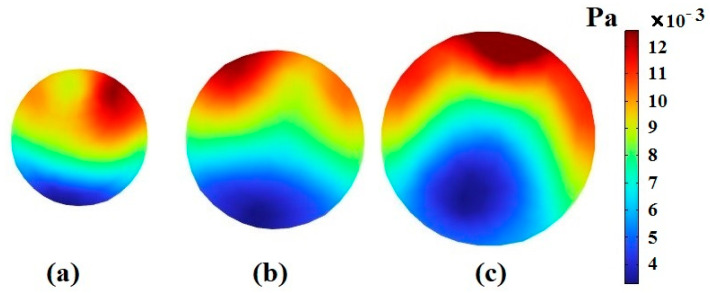
The stress contour inside CTC1 when it reaches the end of the 0.5-loop spiral microchannel when ${Re}_{ch}=1$: (**a**) $r_{P}=6\;\mu m$, (**b**) $r_{P}=8\;\mu m$, and (**c**) $r_{P}=12\;\mu m$.

When the length of the spiral channel and its radius are not significantly large, bending effects are ignored. Therefore, the major force that affects the cells is the shear force, which is caused by the motion of fluid on the boundary of the cell. In this case, the difference in the amount of stress applied to the cells depends on their distance from the channel wall and their distance from each other. Cells that are closer to the channel wall are subjected to more stress. For certain kinds of cells, the one with a larger diameter is in more contact with the fluid layer, resulting in more applied shear stress (Figure 6).

### 5.3. Effect of the Number of Loops

**Figure 7:**
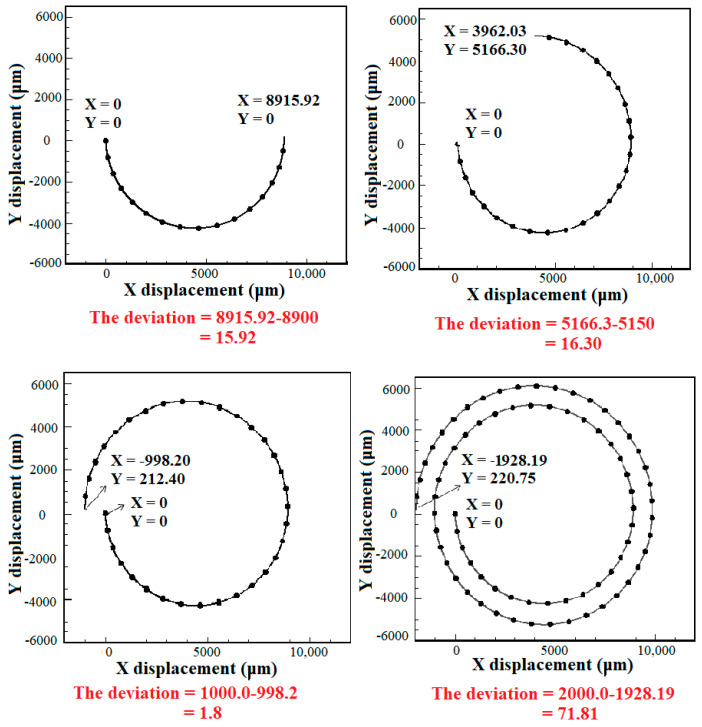
The deviation of CTC1 at the outlet of spiral microchannels with various numbers of loops with respect to the channel centerline when ${Re}_{ch}=1$. The centerline axis at the outlet passes through (X, Y) = (0, 8900), (3962.03, 5150), (−1000, 0), and (−2000, 0) for the 0.5-, 1-, 1.5-, and 2-loop spiral microchannels, respectively. The deviation of CTC1 is calculated utilizing their position and the coordinate of the centerline axis at the microchannel outlet.

In this section, the effect of the number of spiral loops on the hydrodynamic behavior of the deformable cells is evaluated. CTC1 with a diameter of 16 μm along with diluted blood enter the microchannel with various number of loops when ${Re}_{ch}=1$. The number of loops has a great effect on the final position of cells. By augmenting the number of loops, the length of the path that the cells travel is enhanced, reducing their velocity due to an increment in the drag force. Therefore, to choose the optimal number of loops for the isolation of CTCs, the balance between different forces acting on the cells should be considered. Figure 7 depicts the deviation of CTC1 from the channel centerline of different geometries. It is demonstrated that the amount of deviation is 15.92 μm and 16.3 μm toward the outer wall for the 0.5- and 0.75-loop spiral microchannels, respectively. Additionally, the amount of deviation for 1- and 2-loop microchannels is 1.8 and 71.81 μm toward the inner wall, respectively, indicating that the equilibrium position of cells is strongly affected by the lengthening of the microchannel. The increase in the number of loops, i.e., the increment in the radius of curvature, leads to a higher Dean number $De={Re}_{ch}\sqrt{D_{h}/2R}$, which causes the cell to deviate more toward the inner wall. For the case of the 2-loop microchannel, the deviation of CTCs toward the inner wall is much higher than the 0.5-, 0.75, and 1-loop microchannels due to the larger radius of curvature and formation of stronger Dean vortices. Hence, it can be concluded that the 2-loop spiral microchannel can be selected as the optimal case for isolating single cells.

**Figure 8:**
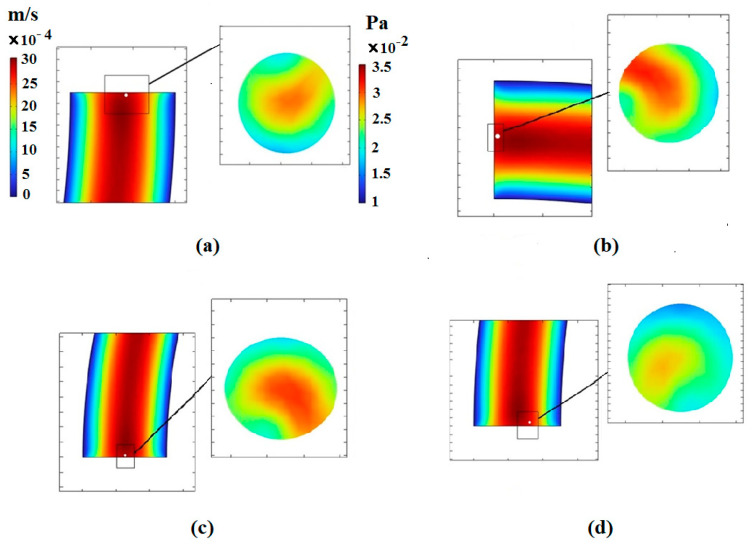
Velocity and stress contours resulting from fluid effects on the CTC1 at the end of the microchannels when ${Re}_{ch}=1$: (**a**) the number of loops is 0.5, (**b**) the number of loops is 0.75, (**c**) the number of loops is 1, and (**d**) the number of loops is 2.

The amount of stress exerted on the cells by the fluid is illustrated in Figure 8. Since the increase in the number of loops affects the pressure and velocity of the fluid, the change in the shape of the cells in microchannels is significant. By augmenting the number of loops, the length of the microchannel increases, leading to a decrease in the fluid velocity. Thus, the centrifugal force and the magnitude of the shear stress are reduced. Figure 8 also depicts the contour of the velocity distribution at the outlet of the microchannels.

### 5.4. Effect of the Microchannel Depth

**Figure 9:**
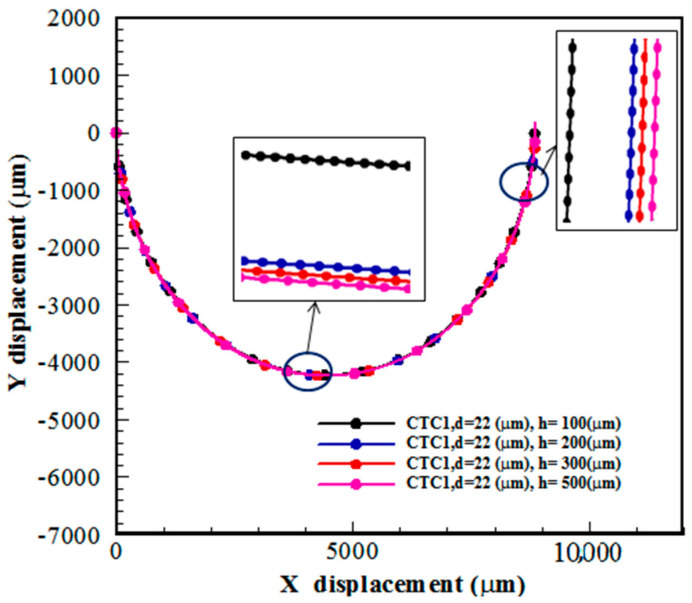
The trajectory of CTC1 in a 0.5-loop spiral microchannel for different amounts of channel depth when ${Re}_{ch}=1$.

The Dean flow in a spiral channel leads to a shorter distance of cells to reach their equilibrium position compared to the straight channel [23]. In this section, the effect of the depth of the spiral microchannels is discussed for CTC1 with a diameter of 22 μm when ${Re}_{ch}=1$. Four values of the channel depth, h = 100, 200, 300, and 500 μm, are considered. Figure 9 shows that the channel depth (channel aspect ratio) has a significant effect on the isolation of CTCs. One of the important parameters for focusing microparticles/cells in microchannels is the blockage ratio, which is defined as the ratio of the cell diameter to the channel hydraulic diameter, $\beta ={2r}_{p}/D_{h}$. It was known that β>0.07 is required to focus microparticles/cells [30]. At β<0.07, the influence of $F_{L}$ on the focusing process is diminished. The amount of β is 0.132, 0.077, 0.058, and 0.044 for h = 100, 200, 300, and 500 μm, respectively, when $r_{p}$ = 11 μm. It should be pointed out that $F_{D}$ and $F_{D-L}$ are two other significant forces that affect the trajectory of cells. In this section, the deformability of CTCs is maintained constant. Since the amount of ${Re}_{ch}$ and the channel width are kept constant, the fluid velocity is reduced by enhancing the channel depth (or $D_{h}$). Thus, the centrifugal force caused by the blood flow is decreased with h, reducing the amount of $F_{D}$. In other words, there is a competition between $F_{L}$ and $F_{D}$ during the motion of CTCs in spiral microchannels. Figure 9 demonstrates that the CTC deviation is augmented with h. This is due to the smaller effect of centrifugal force, i.e.,$\;F_{D}$, on CTC trajectories compared to $F_{L}$.

## 6. Conclusions

Spiral microchannels with various loops are designed and analyzed to isolate deformable CTCs utilizing an ALE and FEM numerically. The trajectory of CTCs is controlled by their deformability and size, number of loops, and channel depth. The present simulations suggest that an inertial-based microfluidic device can be employed to sort deformable cells in clinical systems. It is demonstrated that the cells move toward the outer wall of the microchannel as deformability is augmented. Additionally, larger and smaller deformable cells are directed toward the inner and outer channel walls, respectively, due to the balance between $F_{L}$, $F_{D}$, and $F_{D-L}$. The results reveal that the 2-loop spiral microchannel can be considered the optimal geometry. Furthermore, the microchannel aspect ratio affects the trajectory and deviation of deformable cells. Since diluted blood samples are employed for practical applications, the combination of micromixers [31,32,33] and microfluidic separation devices is recommended for the isolation of CTCs from blood cells. Additionally, inertial focusing of non-spherical deformable cells can be considered to examine the influence of cell shapes on their isolation mechanisms.

## Figures and Tables

**Table 1:** Properties of CTCs and WBCs [26].

	$R_{s}$	$E_{s}$, kPa	$r_{P}$, μm
CTC1	0.4	1000	11
CTC2	10^−8^	1	8
WBC	10^−9^	0.1	6

## Data Availability

Data are contained within the article.
